# Human immunodeficiency virus positive status disclosure to a sexual partner and its determinant factors in Ethiopia: a systematic review and meta-analysis

**DOI:** 10.1186/s12879-020-05081-9

**Published:** 2020-05-29

**Authors:** Fikadu Yehualashet, Eleni Tegegne, Mekbib Tessema, Mulualem Endeshaw

**Affiliations:** 1 Department of community health Nursing, School of Nursing, College of Medicine and Health Sciences, University of Gondar, Gondar, Ethiopia; 2 Leshimaniasis Research and treatment Center, University of Gondar Hospital, Gondar, Ethiopia; 3 Rift Valley University, Addis Ababa, Ethiopia

**Keywords:** HIV/AIDS, Disclosure status, ART, Systematic review and meta analysis, PLWHA, Ethiopia

## Abstract

**Background:**

Disclosure of Human Immunodeficiency Virus positive status significantly reduced the transmission of HIV; yet, it remains a challenge for many HIV patients. Disclosure serves plays a crucial role to raise awareness and to reduce risky behaviors. Hence, this study aimed to determine the pooled prevalence and effect sizes of determinant factors of HIV positive status disclosure through a systematic review and meta-analysis of the results of the existing primary studies in Ethiopia.

**Method:**

This systematic review and meta-analysis was aimed to determine prevalence of HIV positive status disclosure and associated factors by considering and searching published primary articles from different sources. A sensitivity test was conducted to evaluate the presence of influential studies. Besides, the heterogeneity test has been conducted; and publication bias was examined through observing the funnel plot as well as objectively by interpreting the Egger’s regression test. Following the Egger’s regression test, *P*-value < 0.05 was considered as statistically significant at 95% Confidence Interval.

**Result:**

A total of 18 primary studies were searched from different data sources. The overall pooled prevalence of HIV positive status disclosure among adult PLWHA in Ethiopia was indicated to be 75.95% (95% CI:69.93–81.98); the highest and lowest pooled estimated HIV status disclosure was in Amhara (82.78%) and Tigray (54.31%) regions respectively. Furthermore, Knowing the HIV positive status of sexual partner, AOR = 19.66(95% CI: 10.19–37.91), having prior discussion about HIV testing with their partner, AOR = 9.18(95% CI: 5.53–15.24), got Human Immunodeficiency Virus pretest counseling service AOR = 4.29(95% CI: 2.56–7.21) and being a member of HIV/AIDS associations, AOR = 3.34(95% CI: 2.17–5.12), were significantly associated with HIV positive status disclosure among People living With HIV/AIDS in Ethiopia.

**Conclusion:**

The pooled national estimate of HIV/AIDS positive status disclosure is low as compared to the WHO disclosure rate of developing countries and the findings of other national and international studies. Ministry of health and other stakeholders shall design new approaches and strategies to encourage disclosure of HIV status, educate the public about the negative impact of nondisclosure within family members. Health care providers working at Human HIV test centers shall emphasis extensive counseling on disclosure of status to a partner. Moreover, different stakeholders, health workers and community members shall establish, organize, and support HIV/AIDS Associations and motivate HIV positive people to be engaged and participated.

## Background

Globally, about 36.7 million people living with HIV in 2015 which higher than that of 33.3 million in the year 2010. These escalated number of PLWHA was due to the continuing of new infections and increased longevity of PLWHA by Anti Retro Viral Therapy drugs [1]. Sub-Saharan Africa remains most severely affected, with nearly 1 in every 20 adults (4.9%) living with HIV and accounting for 69% of the people living with HIV worldwide [2].

Disclosure is a personal and intimate process which engages the soul, the mind and the body; and might affect self-image, self-efficacy, self-perception, and confidence. Therefore making the PLWHA to be more vulnerable for various forms of psychosocial problems [3]. Self-disclosure of sensitive information is generally thought to have beneficial effects on an individual’s health [4, 5], it lowers stress, and lead to better psychological wellbeing. In regard to HIV/AIDS, individuals who disclose their status are in a better position in terms of reproductive choices, treatment adherence as well as psychosocial support [5]. Disclosure to significant others would provide emotional and psychological support to PLWHAs, since disclosure creates the awareness of HIV risk to untested sexual partners, it subsequently leads to greater uptake of HIV Testing and Counseling (HCT) [6]. Disclosure of HIV test results to sexual partners is associated with less anxiety and increased social support among many women. Furthermore, HIV status disclosure may lead to improved access to HIV prevention and treatment programs, increased opportunities for risk reduction and plan for the future [6, 7]. Disclosing or sharing of HIV status with a sexual partner is encouraged and is an integral practice both in Voluntary counseling and Testing (VCT) and Prevention of Mother to child transmission (PMTCT) programs [8] and freedom to use Antiretiro Viral (ARV) medications [9]. Though it is complex and challenging [10] and failure to disclose can place their sex partners at risk of acquisition of HIV/AIDS [4].

The rate of disclosure in studies from developing countries was notably lower than rates reported from the developed nations. The rates ranged from 16.7 to 86%. Among the studies that reported the average rate of disclosure to current sexual partner was 49%, considerably less than the average rate (79%) reported from studies conducted in developed countries [2]. Disclosure of HIV positive status is an important part of coping with the disease and understanding the surrounding circumstances and is critical in both the prevention of HIV and mitigation of its impact [11]. Human Immuno Virus positive status disclosure is associated with several advantages such as improved drug adherence, social support, and a better physical as well as psychological wellness [8, 12]. Recent evidences suggests that, disclosure is more common to primary sex partners than to non-primary sexual partners and to HIV-infected partners than to HIV-negative or unknown HIV status partners [4]. A study conducted in the Ogun state of Nigeria indicated that 50.9% of study participants have disclosed their HIV status to their main sexual partner [5].

Several factors are associated with disclosure of HIV status to sexual partner these include but not limited to: fear of the negative outcomes of disclosure, communication barrier to disclose, having initiated antiretroviral therapy, participating in HIV associations, and having ever seen people publicly disclose their HIV status [8].

A study conducted in Jimma University Hospital, Ethiopia, showed that living in the same house, prior discussion, knowing HIV status of partner, clinical stage and level of negative self-image contribute to HIV status disclosure. On the contrary, fear of separation or divorce, not to worry about partner, fear of accusation of infidelity, fear of physical abuse, fearing that partner will be angry and fear of partner getting HIV from me were identified as the main reasons for not disclosing HIV Status [13].

Ethiopia has been devising many efforts to control HIV through various strategies like provision of ART, PMTCT, offering extensive adherence counseling and expanding HIV testing sites and approaches. Besides, it also proposed a new HIV prevention and control program; the three 90s and test and treat approach as per the direction and plan of United States Agency for International Development (USAID). Although multiple primary studies were done in various parts of Ethiopia that pointed out the prevalence of HIV positive status disclosure as well as the determinant factors among people living with HIV, these all findings are reported in a contradicted and inconclusive way. Therefore, this study is aimed to determine the pooled prevalence and determinants of HIV status disclosure among people living with HIV in Ethiopian. Understanding of the national pooled estimate and determinant factors of disclosure could enable researchers, clinicians, policymakers, HIV programmers to evaluate the existing strategies as well as to establish new HIV prevention and control projects and might help to reduce the spread of HIV infection in the community.

## Methods

### Protocol registration and review reporting

The developed protocol has been registered at the international prospective register of Systematic Review and meta-analysis (PROSPERO) and received a registration number of CRD42019116701. The Preferred Reporting Items for Systematic Reviews and Meta- Analyses (PRISMA) checklist and the Meta-analysis Of Observational Studies in Epidemiology (MOOSE) were also used to present the methods as well as the results of the study [14, 15]. All selected primary studies were qualitatively described in terms of the different variables such as; Authors name, prevalence, sample size, study area, study period, population, Standard error of prevalence and Odds ratio. Screening and selection procedures of the eligible studies were illustrated through the Joanna Briggs Institute **(**JBI) Quality Assessment tool. Articles that scored more than 50% of the JBI Quality Assessment were eligible for the review. Moreover, the results of the study were presented using tables, texts, and figures accordingly.

### Data source and search strategy

To identify a sufficient number of primary articles different data sources such as PubMed/Medline, Health Inter Network Access to Research Initiative (HINARI), and Scopus were explored. More articles have been retrieved using the electronics search engines of Google and Google scholar. All references of the relevant articles have been used to access additional studies. During the searching process, just to diminish irrelevant studies, searching was restricted to only “human studies” and done in “English language”. Corresponding authors have contacted via mail or other means of communication to access full texts of studies. The key search terms were “HIV disclosure status”, “HIV infection”, “Disclosure”, “Seropositive”, “determinant factors”, “associated factors”, “People living with HIV”, “sexual partner”, “Adherence”, and “Ethiopia”. All these terms were searched in advance search of databases through “MeSH terms” and “All fields” by linking “AND” and “OR” Boolean operator terms as appropriate. Furthermore, Ethiopian Universities’ (Gondar, Haremaya, Jimma and Addis Ababa) online repository library were searched to access additional articles. These all processes of searching were completed by December 20/2018.

### Outcome measurement

- HIV status disclosure is informing own HIV positive result to a sexual partner [3, 4, 12]. It has been mentioned several times in the included studies.
- Sexual Partner: Refers to two people who have sex relation together as husband and wife, boyfriend and girlfriend [4].

### Eligibility criteria

#### Inclusion criteria

All studies conducted to investigate HIV positive status disclosure to sexual partners and determinant factors among people living with HIV in Ethiopia which was:

- Primary studies
- Observational studies, including cross-sectional and analytical cross-sectional
- Written in the English language
- Articles done before this review were included under the review.

### Exclusion criteria

- Studies done to show the HIV positive status disclosure of children
- Qualitative studies
- Observational studies such as experimental studies
- Studies with limited access of full text.

### Quality assessment

The quality appraisal of the eligible articles has been checked by three independent reviewers (FAY, MKT, and ETT). The Joanna Briggs Institute (JBI) critical appraisal assessment material for analytic cross-sectional [16] and cross-sectional [17] studies was used to evaluate its quality. The tool has ‘Yes’ or ‘No’ response, and ‘1’ and ‘0’ values were given for “yes” and “no” responses, respectively; after that the summation result were changed to percentage. Articles whose JBI score is 50% and above were incorporated into the review. Disagreements between assessors were managed through discussion and majority decisions among the three reviewers.

### Data extraction process

Once after the quality assessment has been conducted data were extracted on excel Microsoft spreadsheet by two independent authors (ET and FA) and cross-checked for its consistency by MK. The data were extracted on the following issues, the first author of the article, year of publication, study area, study region, study design, study population, sample size, the prevalence of HIV positive status disclosure, response rate, and its predictors. Any disagreements during the extraction were solved by mutual discussion and consensus (supplementary file 1).

### Data analysis and assessment of publication Bias

The extracted data on Microsoft Excel Database were imported into Software for Statistics and Data Science (STATA) version 11 for analyses. Meta-analyses were performed separately for each outcome. A weighted inverse variance random-effects [18] were used to estimate the overall pooled prevalence of HIV positive status disclosure to sexual partners among adults living with HIV and its determinant factors. Subgroup analysis by region and year of study was done to estimate regional variations of HIV status disclosure prevalence and to point out the trend of HIV status disclosure prevalence by categorizing study periods into three. The percentage of total variation between studies due to heterogeneity was detected using I^2^ [19]. I^2^ test value of 25, 50, and 75% were affirmed as low, moderate and high level of heterogeneity respectively [19]. Furthermore, test for Heterogeneity among studies was achieved using Cochran’s Q statistical test. Publication bias was checked subjectively through observing the funnel plot and objectively through Egger’s regression test. Hence, statistical significant publication bias was declared at a *p*-value less than 0.05 at 95% CI.

## Result

### Study screening and selection processes

**Fig. 1:**
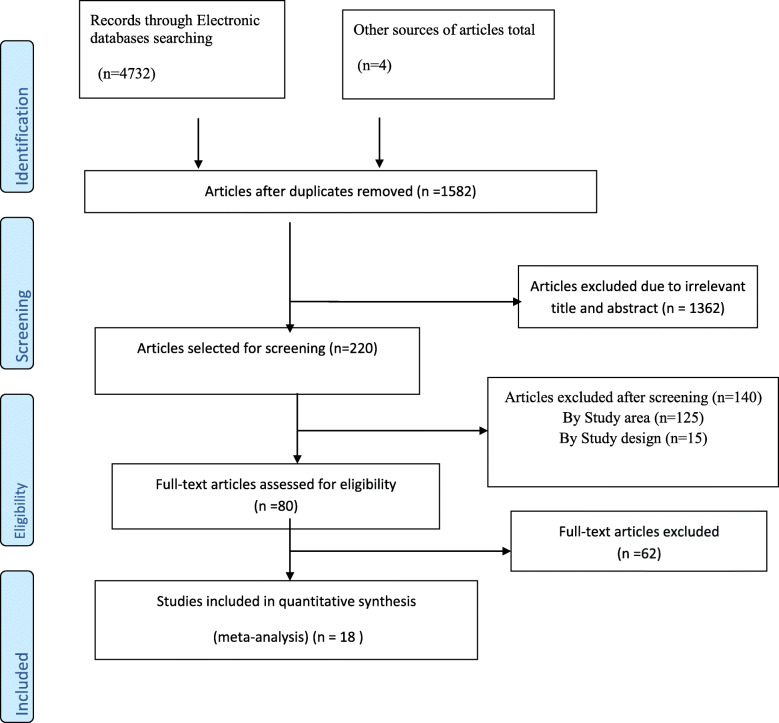
Flow diagram showing studies screened, assessed for eligibility, and included in the review

About 4736 articles were searched from different data sources. Of which, 29 articles from PubMed, 129 articles from Google scholar, 2094 from HINARI, 2480 from Google, and 4 articles from university repositories. However, 3154 articles due to duplicates; 1362 articles due to irrelevant titles and abstracts; 140 articles due to study setting and design were removed. About 80 articles were selected for full-text review. Of these, 62 articles were excluded after full-text review. Finally, 18 studies were included in this systematic review and meta-analysis to estimate HIV positive status disclosure and identify the determinant factors of HIV positive status disclosure among PLWHA in Ethiopia (Fig. 1).

### Characteristics of the included studies

In this systematic review and meta-analysis, a total of 18 articles with a total of 7084 study participants were included. Regarding the region/location of studies conducted, about one third of the studies were from Oromia region [1, 20–24], Amhara region accounts the second largest segment of the included studies 22.22% [13, 25–27], Tigray region has contributed three studies [2, 8, 28], two regions SNNPs [29, 30] and Addis Abeba [31, 32] each supplied a couple of studies and one study was found from Hareri region [33]. Regarding the study design all the included studies were cross-sectional. Concerning the JBI assessment nineteen studies were review and the highest score was 100% which has occurred among six studies [2, 13, 20, 23, 25, 29, 32] and the lowest value was 33.33% from a study conducted in Metu and Gore towns Illubabur Zone Southwest Ethiopia [34] which is subjected to exclusion from the review; However, from the included studies the lowest value was 66.66% among two articles [26, 31]. In addition to this, the lowest and the highest response rate of the included studies was between 95.5 and 100. The highest prevalence of HIV positive status disclosure among people living with HIV was reported in Amhara region, Kemissie zone (93.1%) [13] and the lowest was from Oromia region, Bale Zone (52%) [24]. Based on the JBI quality assessment the average quality of the included studies was 62%. Nineteen articles were involved into the systematic review and Meta-analysis as their JBI score is 50% and above. One study conducted in Gore and Metu towns Oromia region [35] has been excluded from the review as it accounts about 37.5% of the JBI quality assessment (Table 1). Moreover, JBI quality assessment for studies reviewed to determinant factors affecting HIV positive status disclosure has been performed and almost all studied scored more than 80%.

**Table 1:** General characteristics of included studies that report the prevalence of HIV positive disclosure status and its determinant factors

Author	Year of study	Region	Study population	Sample size	P (%)	logp	logSE	LBP	UBP	JBI QA
Alema H et.at [2]	2013	Tigray	Adult PLWH	361	41.80	3.73	2.59	36.71	46.89	100%
AlemayehuM et.al [28]	2013	Tigray	Women PLWH	315	63.80	4.15	2.70	58.49	69.11	77.77%
Bedilu D et.al [29]	2017	SNNPE	Women PLWH	207	72.90	4.28	3.08	66.84	78.96	100%
Binega F et.al [25]	2017	Amhara	Adult PLWH	362	71.00	4.26	2.38	66.32	75.67	100%
Daniel A et.al [26]	2013	Amhara	Women PLWH	263	89.70	4.49	1.87	86.02	93.37	66.66%
Endalew G et.al [31]	2011	Addis Abeba	Women PLWH	107	73.00	4.29	4.29	64.58	81.41	66.66%
Erku T et.al [27]	2010	Amhara	Adult PLWH	334	76.80	4.34	2.30	72.27	81.33	77.77%
Gadisa T et.al [20]	2013	Oromia	Adult PLWH	686	91.10	4.51	1.08	88.96	93.23	100%
Gari T et.al [30]	2008	SNNPE	Women PLWH	384	85.70	4.45	1.78	82.19	89.2	88.90%
Genet M et.al [8]	2012	Tigray	Adult PLWH	324	57.40	4.05	2.74	52.01	62.78	88.88
Getinet K et.al [21]	2017	Oromia	Women PLWH	337	83.00	4.41	2.04	78.98	87.01	88.88%
Kassaye K et.al [22]	2007	Oromia	Adult PLWH	640	90.20	4.50	1.17	87.89	92.5	77.77%
Noah G et.al [32]	2015	Addis Abeba	Adult PLWH	676	82.50	4.41	1.46	79.63	85.36	100%
Reda A et.al [33]	2010	Harer	Adult PLWH	606	66.30	4.19	1.92	62.53	70.06	88.88%
Seid M et.al [13]	2009	Amhara	Adult PLWH	360	93.10	4.53	1.33	90.48	95.72	100%
Shewaye F et.al [23]	2013	Oromia	Adult PLWH	360	84.90	4.44	1.88	81.20	88.6	100%
Tesfaye T et.al [1]	2014	Oromia	Adult PLWH	351	33.33	3.50	2.51	28.39	38.26	88.88%
Tsige D et.al [24]	2017	Oromia	Adult PLWH	411	52.60	3.96	2.46	47.77	57.43	88.88%

### Prevalence of HIV positive status disclosure

**Fig. 2:**
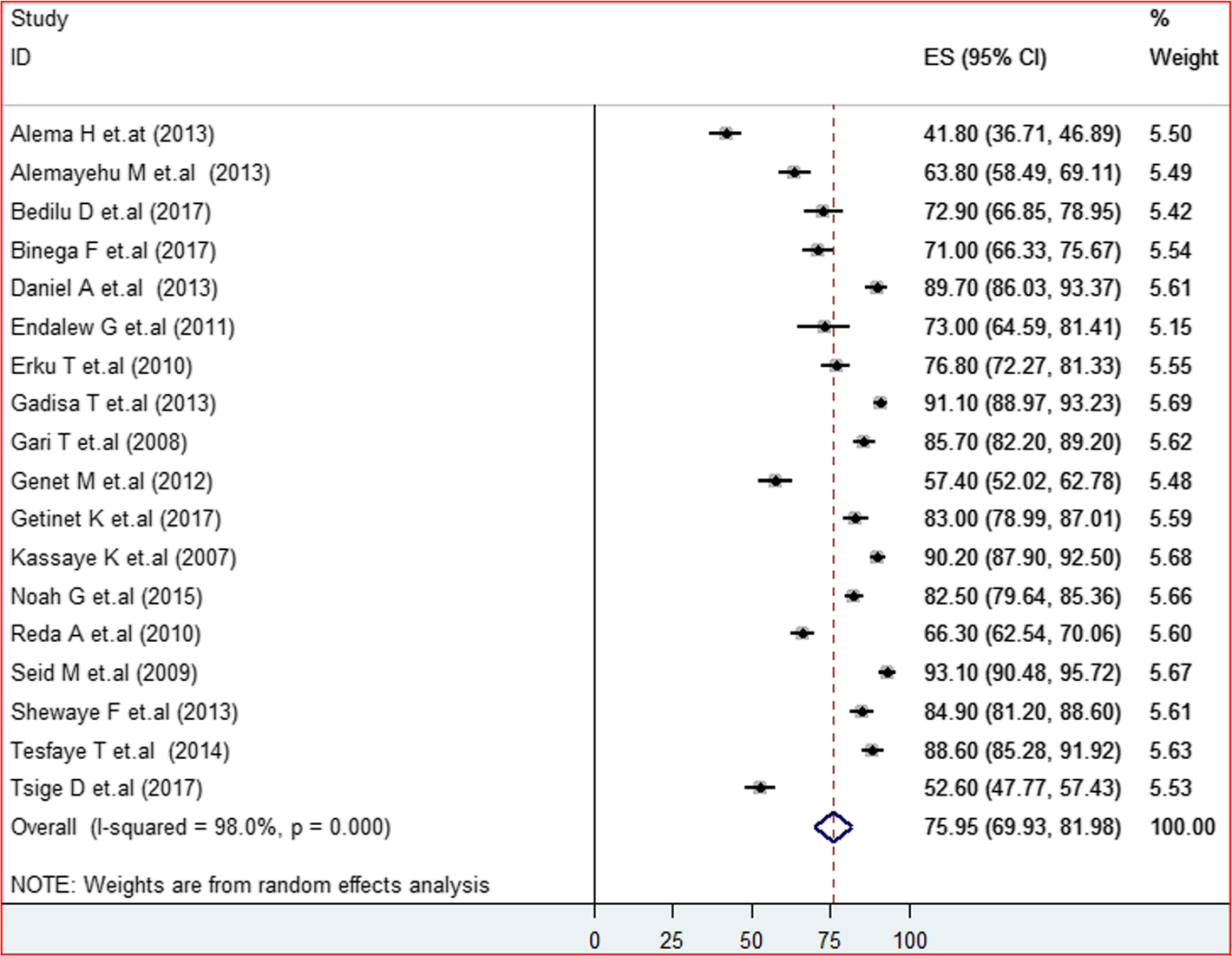
Forest plot of the pooled prevalence of HIV positive status disclosure to sexual partners among adult people living with HIV in Ethiopia at 95%CI

In the random-effects model, HIV positive status disclosure of people living with HIV in Ethiopia was found 75.95% (95% CI, 69.93–81.98%, I^2^ = 98%, P < 0.001) (Fig. 2.).

### Subgroup analysis

**Fig. 3:**
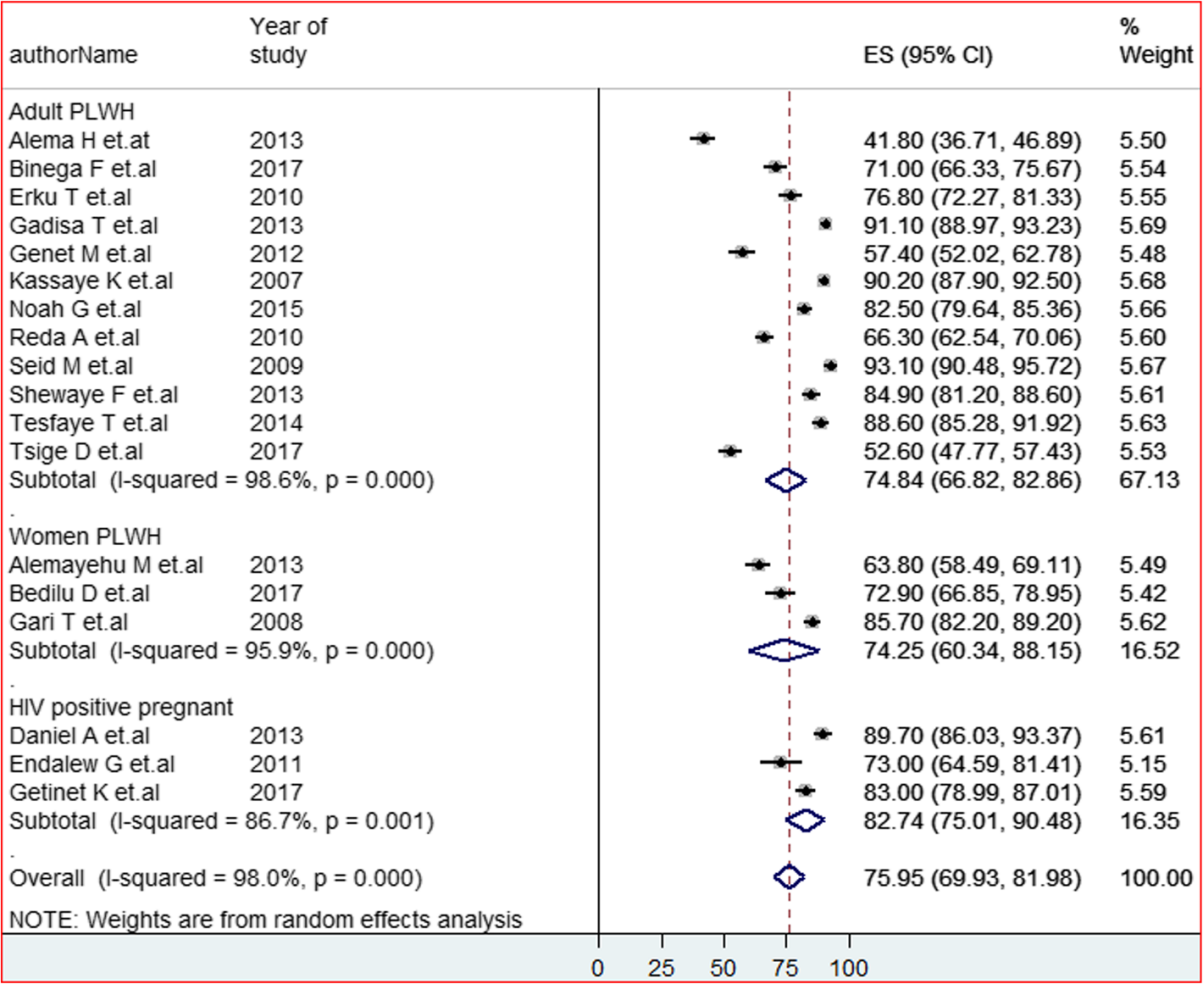
Forest plot of the pooled prevalence of HIV positive status disclosure to sexual partners of a different segment of PLWHA in Ethiopia at 95%CI, the midpoint of each line illustrates the prevalence rate estimated in each study

**Fig. 4:**
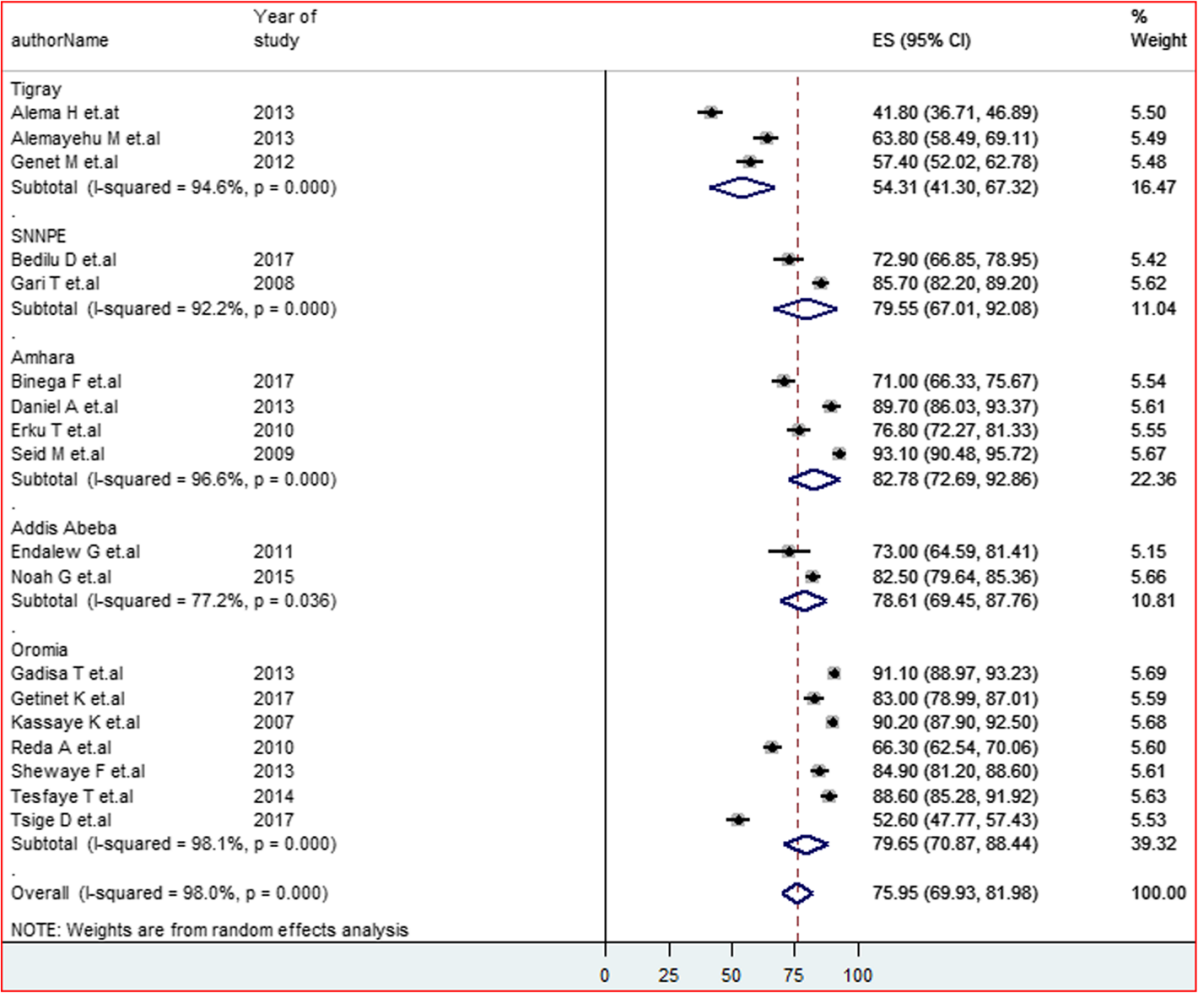
Forest plot of the pooled prevalence of HIV positive status disclosure to sexual partners of different regions among PLWHA in Ethiopia at 95%CI, the midpoint of each line illustrates the prevalence rate estimated in each study

Subgroup analysis has been carried out by the type of population and regions of the studies. As a result, the output of the subgroup analysis with three categories of the population such as ART users, women on ART and HIV positive pregnant women attending the PMTCT care unit revealed a significant difference has been observed between PMTCT users (82.74%) and ART users. However, there was no difference between the adult PLWHA (74.84%) and women on ART (74.25%) (Fig. 3). On the other hand, noticeable variation among study regions has been detected. The highest and lowest pooled estimated HIV status disclosure was at Amhara region (82.78%) and Tigray region (54.31%) respectively (Fig. 4).

#### Publication bias test

**Fig. 5:**
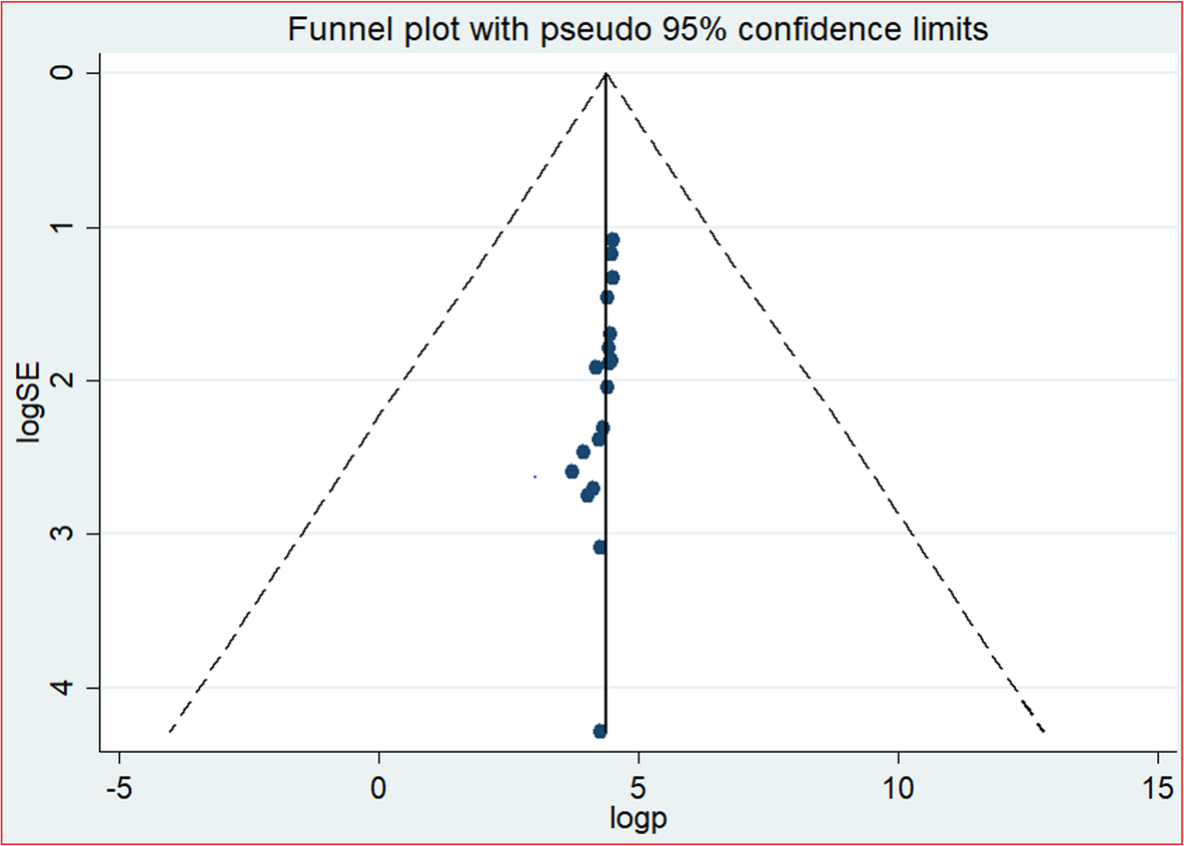
Funnel plot, in which vertical line indicates the effect size whereas diagonal line indicates precision of individual studies with 95% CI, 2019

**Fig. 6:**
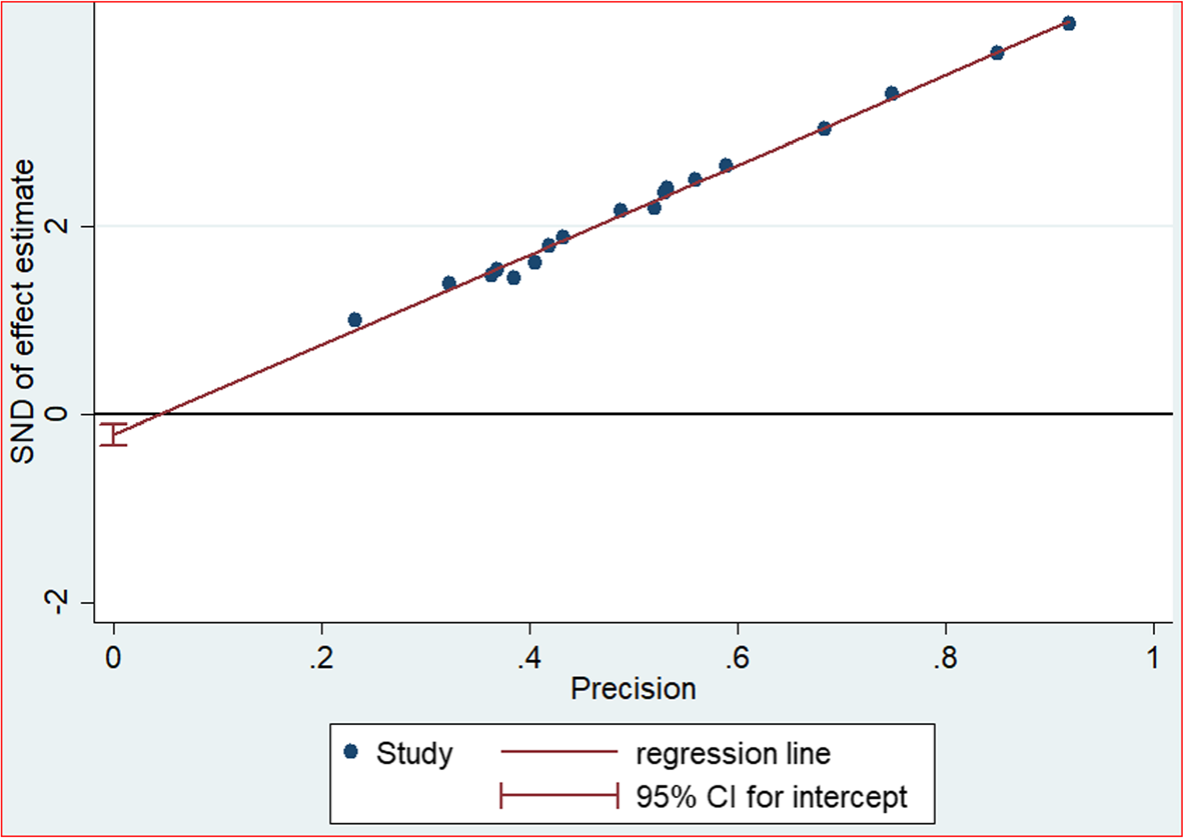
Egger graph showing publication bias among studies conducted to determine HIV status disclosure in Ethiopia, 2019

The presence of publication bias was evaluated both subjectively as well as objectively. The symmetrical alignment of studies in the funnel plot (Fig. 5) and eggers regression graph (Fig. 6) revealed the absence of publication bias among the included studies. However, the statistical value of eggers regression test proved the presence of publication bias *(P = 0.001).*

### Sensitivity analysis

**Fig. 7:**
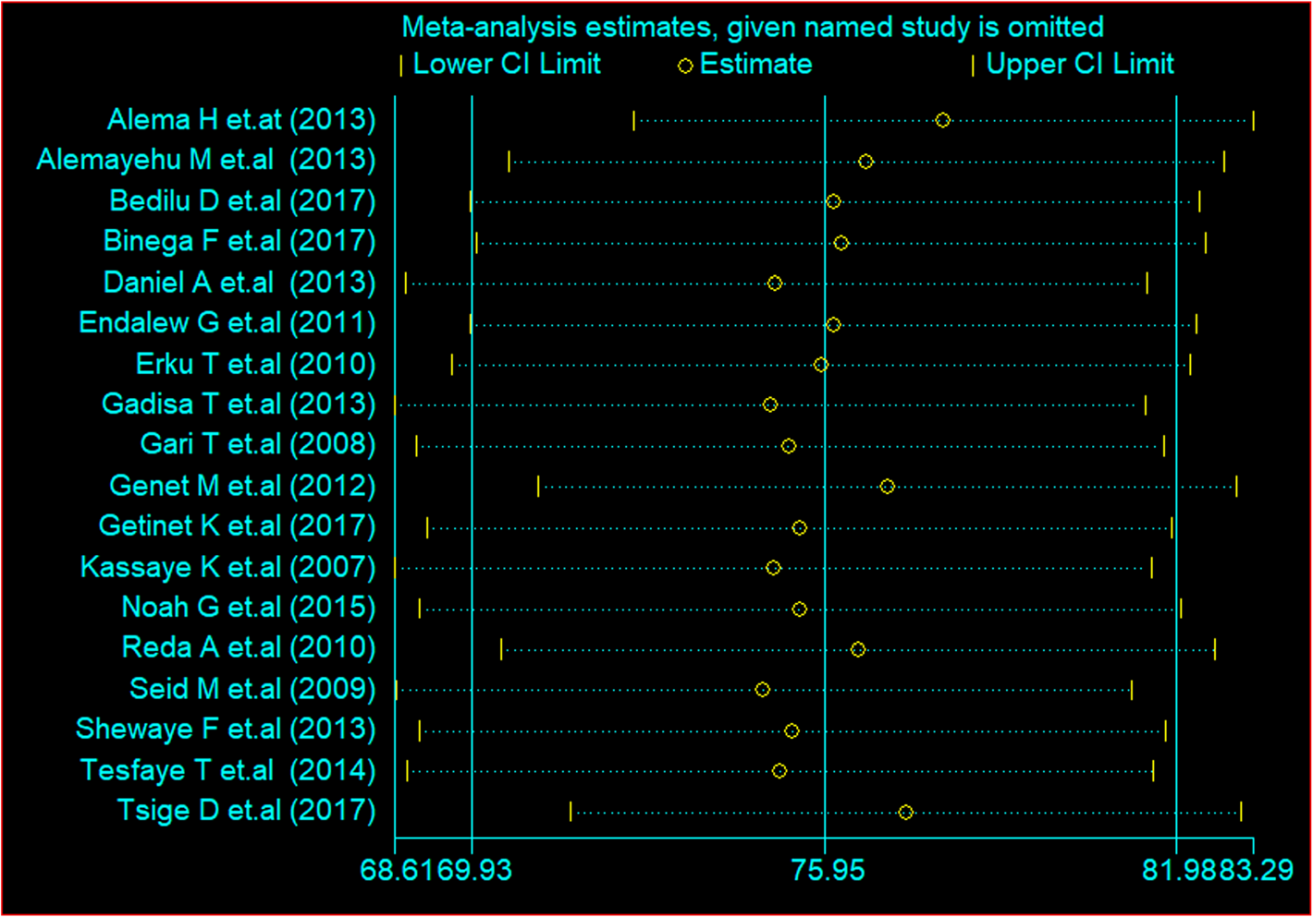
Sensitivity analysis showing presence of influential study among studies conducted to determine HIV status disclosure in Ethiopia, 2019

There was no influential study that affects the pooled estimate of HIV positive status and caused disparity between studies, according to the sensitivity analysis (Fig. 7).

### Determinant factors of HIV positive status disclosure to sexual partner

**Fig. 8:**
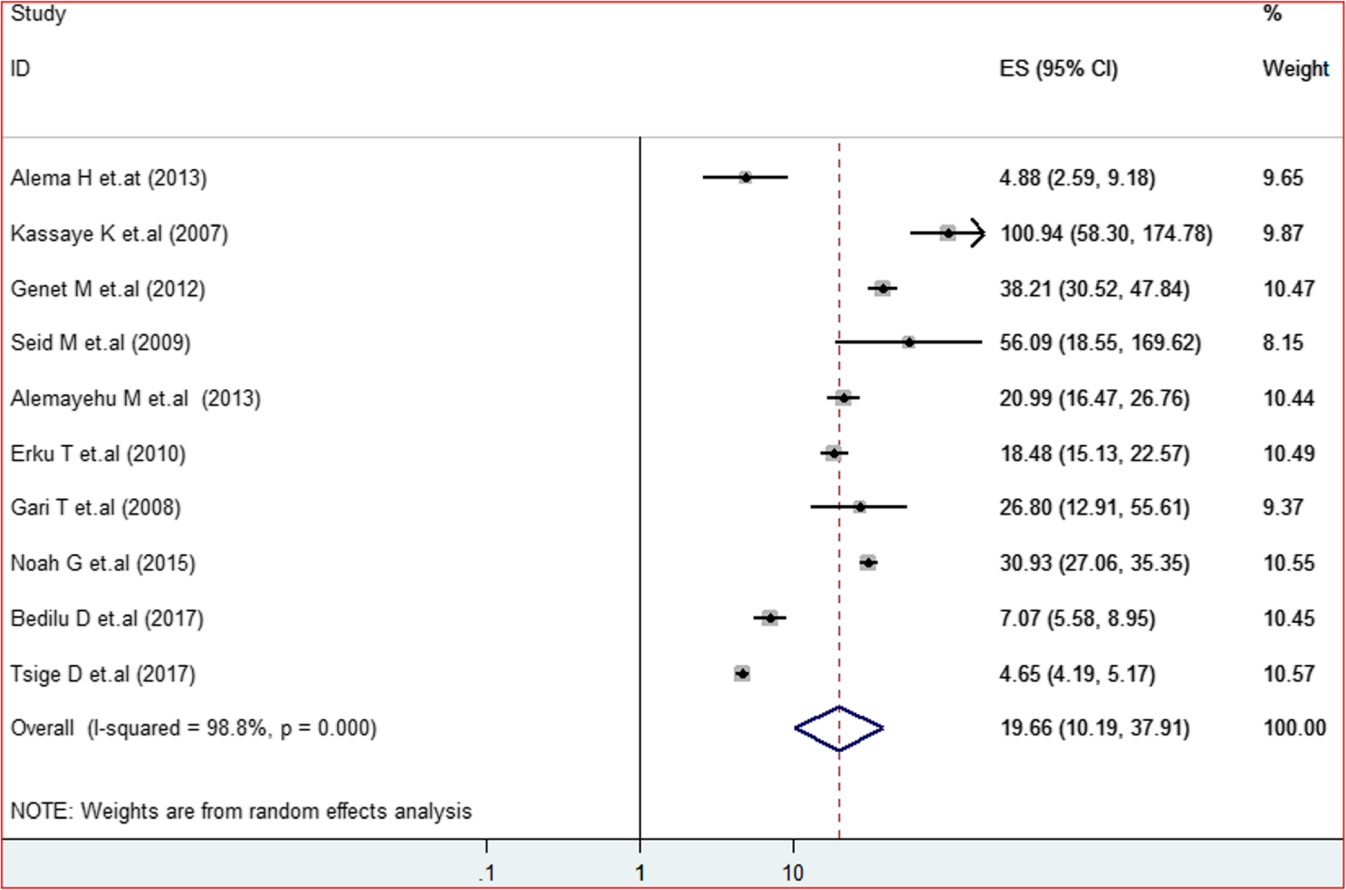
Forest plot of the adjusted odds ratios with corresponding 95% CIs of studies on the association of HIV status disclosure and knowing HIV status of sexual partner

There were a total of ten studies that reported the effect of knowing the HIV status of sexual partner towards HIV status disclosure of PLWHA. Of these, three of the studies were from Tigray region [2, 8, 28]. Amhara [13, 27], Oromia [22, 24] and SNNPs [29, 30] each contributed a couple of studies and one study was from Addis Abeba [32]. All the included studies demonstrate a significant association with the HIV status disclosure. About 70% of the involved studies showed an AOR greater than 10. Hence knowing HIV positive status of a sexual partner was almost 20 times more likely to disclose their HIV test result to their sexual partner than those who didn’t know the status of their sexual partner 19.66(10.19,37.91; I^2^ = 98.8%; *P* < 0.001) (Fig. 8). The sensitivity analysis revealed no small study effect that distorts the pooled estimate. Furthermore, both the subjective and objective publication assessment confirmed the absence of bias (*P*-value = 0.297).

**Fig. 9:**
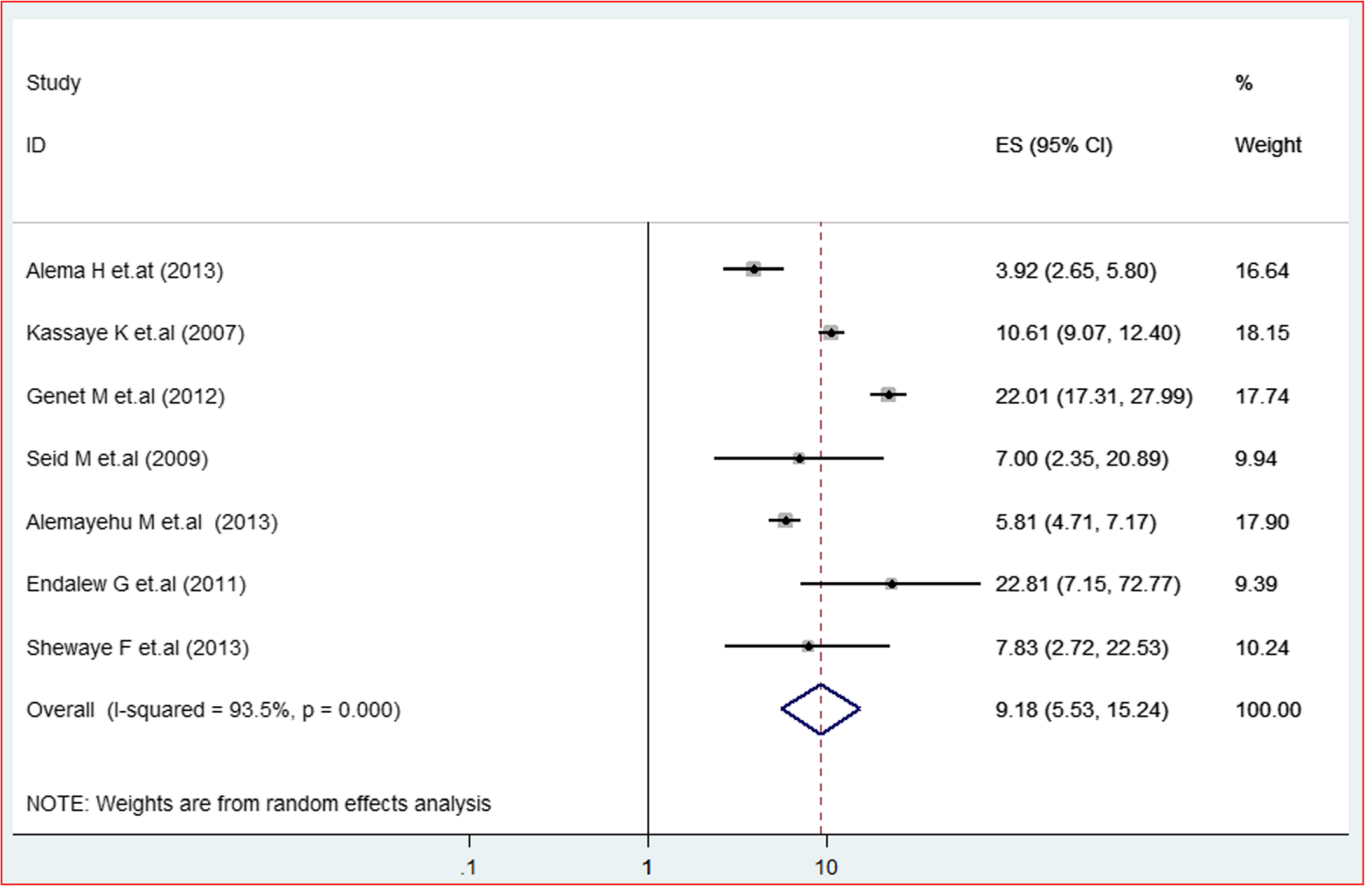
Forest plot depicting the interaction between presence of prior discussion about HIV testing with sexual partner with HIV status disclosure at 95% CIs a systematic review and meta analysis in Ethiopia, 2019

The second outcome variable tested to verify the presence of association with HIV positive status disclosure was a discussion with a sexual partner before HIV testing. As a result, a total of seven studies from four regions were identified reporting effect of prior discussion about HIV testing with a sexual partner with HIV positive status disclosure. From these, three of these studies were from Tigray region [2, 8, 28], two from Oromia region [22, 23] and Addis Abeba [31] and Amhara region [13] each contributed a single study. Of the included studies one study [2] does not show an association between having prior discussion about HIV testing with a partner and HIV positive status disclosure. The rest of the studies showed a positive association with a minimum AOR of 2.99 [13] and a maximum AOR value of 12.28 [31]. Hence, the likely hood of disclosing HIV positive status to their sexual partner among clients who had prior discussion about HIV testing was nine-fold higher than their counterparts 9.18(95% CI = 5.53, 15.24; I^2^ = 93.5%; *P* < 0.001) (Fig. 9). The effect of small studies has not been observed. Furthermore, the eggers regression test revealed the absence of publication bias (*P* = 0.852).

**Fig. 10:**
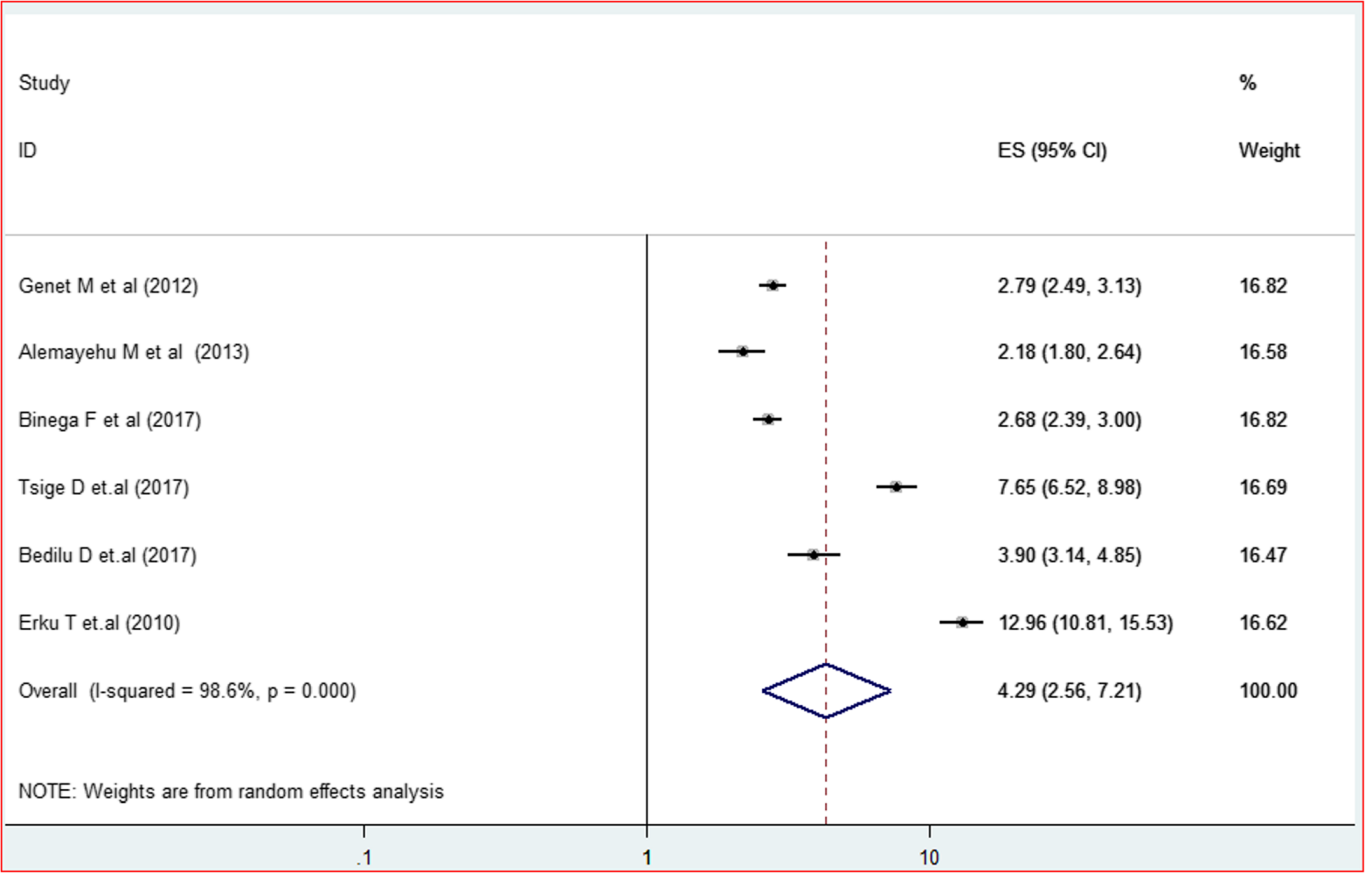
Forest plot showing effect of HIV counseling service on HIV positive status disclosure with corresponding 95% CIs, a systematic review and meta analysis in Ethiopia

The third variable identified to examine the presence of association was getting pre-test HIV counseling. Six primary studies that showed the effect of HIV counseling on HIV positive status disclosure were identified and reviewed. Amhara [25, 27] and Tigray [8, 28] regions each contributed two studies, while Oromia [24] and Southern Nations Nationalities and Peoples SNNPs [29] each added a single study to the review. Originally, getting pretest counseling service demonstrated a significant positive association with HIV positive status disclosure with an AOR ranging between 2.8 and 6.25. On top of this, the meta-analysis revealed that PLWHA who got pretest counseling service has about four times higher chance of disclosing their HIV positive status to their sexual partner/s 4.29(95% CI = 2.56, 7.21; I^2^ = 98.6%; *P* < 0.001)(Fig. 10). Moreover, the sensitivity analysis and eggers regression test testified the absence of influential study and publication bias (*P* = 0.349).

**Fig. 11:**
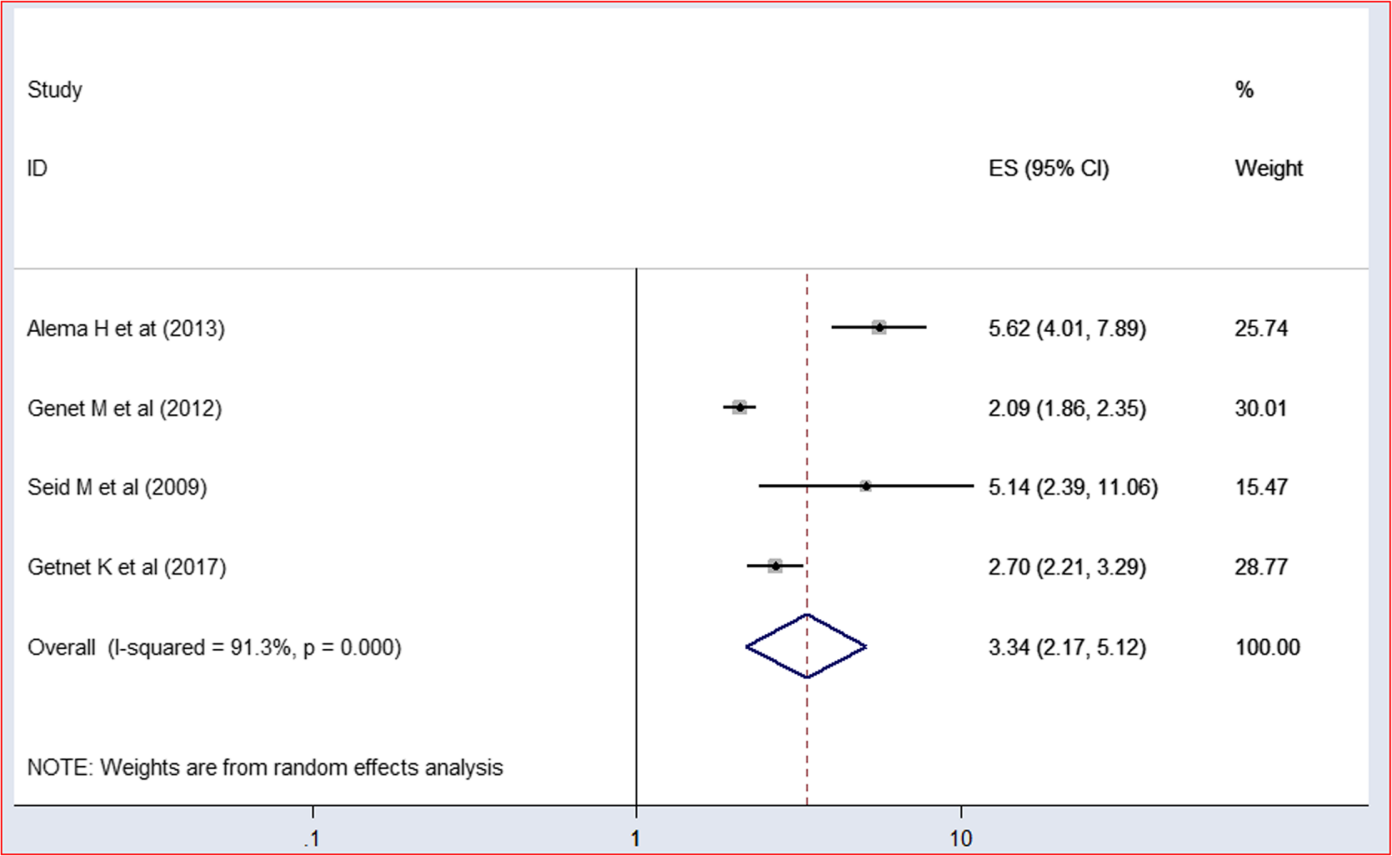
Forest plot showing the effect of Membership in the HIV/AIDS associations on HIV positive status disclosure among PLWHA with corresponding 95% CIs, a systematic review and meta analysis in Ethiopia

Lastly, this systematic review and meta analysis (SRMA) also evaluates the effect of participation in the HIV Association on HIV positive status disclosure. Hence, about four articles from three regions; two from Tigray region [2, 8], one from Amhara [13] and the other from Oromia [21] regions were analyzed. Each study independently showed a significant association with an odd ration ranging between 2.09–5.2. The meta-analysis revealed that PLWHA who are members of HIV associations have more than a threefold chance of disclosing their HIV positive status to their sexual partner/s than their counterparts 3.34(95% CI: 2.17–5.12;I^2^ = 91.3%; *P* < 0.00)(Fig. 11). Furthermore, the sensitivity analysis and eggers regression test testified the absence of influential study as well as publication bias (*P* = 0.147).

## Discussion

HIV/AIDS is one of the most disparaging outbreak the world has ever faced [36]. Disclosing HIV positive status helps not only the individual client but also the society in different perspectives [31]. HIV positive status disclosure has become an important strategy for programs like the prevention of mother to child transmission of HIV, couple HIV counseling and testing and better ART adherence [28, 31, 37]. Notifying the HIV positive status to a sexual partner and other family members allows the client to acquire any preventive, curative as well as rehabilitative support they demand during their life [36]. Studies evidenced that, women who disclose their HIV positive status to their sexual partners are highly liable to engage in the prevention of mother to child transmission (PMTCT) of HIV [31, 38].

Disclosing one’s HIV positive status may, however, expose people to the risks of negative social effects such as stigma, violence, discrimination and rejection [28, 39]. A Meta-analysis study carried out in both industrialized and developing nations revealed a significant variation in the average HIV positive status disclosure rate 79, and 49% respectively [30].

As evidenced by this SRMA, the national pooled estimated prevalence of adult HIV positive status disclosure to sexual partners is 75.95% (95% CI: 69.93–81.98%). This finding is higher than a study conducted in Tanzania 41% [10], Southwestern Nigeria 51% [40], the Ogun State of Nigeria 50.9% [5] and Vietnam 72.9% [41], however, it is lower than a study conducted in Botswana 82% [3], Tanzania 93.3% [42], and a study conducted in HIV clinics of Kenya, Namibia, and Tanzania 80% [43], rural Nigeria 86.5% [44]. This discrepancy might be due to a difference in the HIV prevention programs and the demographic variations of nations.

The present study explored factors that determine the HIV positive status disclosure to sexual partner. As a result, knowing the HIV status of sexual partner has found a strong independent predictor of HIV positive status disclosure. PLWHA, who were aware of the HIV status of their partners were almost 20 times more likely to disclose their status to their spouses. This finding has been supported by studies conducted in African countries like Kenya, Namibia, Tanzania [43], Botswana [3] and a qualitative study done in South Africa [11], as well as a study conducted in Haiti [45]. This could be because knowing partners HIV status may diffuse blame, shame, and fear of conflict with a sexual partner after disclosure.

In addition, having a prior discussion about HIV testing with sexual partners has been identified as a determinant factor that significantly affects HIV positive status of PLWHA. Clients who discussed the issue of HIV testing with their spouses before the test were nine times more likely to disclose their HIV positive status than their counterparts. This finding is cognizant with a study conducted in Tanzania [10]. This could be due to the reason that, couples who discussed about HIV test will be preoccupied about the anticipated result of the test and can easily accept the positive result of their partners. On top of this, prior discussion will help couples to be aware of their HIV risks and expect positive test result. Hence, they will easily accept the test results of their sexual partner when they are informed by them.

On the other hand, being a member of HIV/AIDS associations were another determinant variable affecting HIV status disclosure. The chance of HIV status disclosure to sexual partner has increased by three fold among PLWHA participating in HIV/AIDS associations. PLWHA, who participated in different HIV associations, would be more benefited from the knowledge and experience they shared from colleagues and would be well aware about the process and consequences of disclosure. This finding is evidenced by a study conducted in Brazil [46].

Furthermore, getting HIV pretest counseling services increased the chance of HIV status disclosure to sexual partners. Those clients who got pretest counseling were about four times more likely to disclose their status to their sexual partners than those who didn’t got counseled. This finding was also congruent with a study conducted in Mityana district of Uganda [6] and Southwest Nigeria [40]. One of the major goals of HIV counseling is to encourage clients to disclose their HIV status to their partners as well as their significant others. Effective pretest counseling services will provide clients to develop self-efficacy for the disclosure and provide them knowledge on the importance of partner notification, adherence, disclosure, and other related issues.

## Conclusion

The pooled national prevalence of HIV status disclosure is low compared to different national and international studies. Knowing the HIV status of a sexual partner, conducting prior discussions about HIV testing with partner, being a member of HIV/AIDS Associations and getting HIV Counseling service were found predictors of HIV status disclosure to sexual partner in Ethiopia. Ministry of health shall design new approaches and strategies to encourage disclosure of HIV status, educate the public about the negative impact of nondisclosure within family members. Health care providers working at HIV test centers shall emphasis extensive counseling for HIV positive clients on disclosure of status to partner. Lastly, different stakeholders, health workers, and community members shall establish, organize and support.

## Supplementary information

**Additional file 1.**

## Data Availability

All data and materials are included in the paper.

## Abbreviations

AIDS: Acquired Immunodeficiency Syndrome
AOR: Adjusted Odds Ratio
ART: Anti Retroviral Treatment
CI: Confidence Interval
HINARI: Health Inter Network Access To Research Initiative
HCT: HIV Testing and Counseling
HIV: Human Immunodeficiency Virus
JBI: Joanna Briggs Institute
MOOSE: Meta-Analysis Of Observational Studies In Epidemiology
PLWHA: People Living With HIV/AIDS
PMTCT: Prevention of Mother To Child Transmission
PROSPERO: Prospective Register Of Systematic Review And Meta-Analysis
SNNP: Southern Nations Nationalities and Peoples
PRISMA: Preferred Reporting Items for Systematic Reviews and Meta- Analyses
USAID: United States Agency for International Development
WHO: World Health Organization

## References

[1] Tesfaye T, Darega J, Belachew T, Abera A (2018). HIV positive sero-status disclosure and its determinants among people living with HIV/AIDS following ART clinic in Jimma University specialized hospital, Southwest Ethiopia: a facility-based cross-sectional study. Arch Public Health.

[2] Alema HB, Yalew WA, Beyene MB, Woldu MG (2015). HIV positive status disclosure and associated factors among HIV positive adults in Axum health facilities, Tigray, northern Ethiopia. Sci J Public Health.

[3] Tshisuyi ET. Disclosure of hiv positive status to sexual partners among pregnant women in a health district of botswana. Stellenbosch University; 2014. https://scholar.sun.ac.za/bitstream/handle/10019.1/86431/tshisuyi_disclosure_2014.pdf;jsessionid=C5668FC332FD1BE6678F128DD859BEB1?sequence=2.

[4] OSS MV (2012). Disclosure of HIV status to sexual partners by people living with HIV, South Africa.

[5] Amoran O (2012). Predictors of disclosure of sero-status to sexual partners among people living with HIV/AIDS in Ogun state, Nigeria. Niger J Clin Pract.

[6] Kadowa I (2009). Factors influencing disclosure of HIV positive status in Mityana district of Uganda. Afr Health Sci.

[7] Medley A, Garcia-Moreno C, McGill S, Maman S (2004). Rates, barriers and outcomes of HIV serostatus disclosure among women in developing countries: implications for prevention of mother-to-child transmission programmes. Bull World Health Organ.

[8] Genet M, Sebsibie G, Gultie T (2015). Disclosure of HIV seropositive status to sexual partners and its associated factors among patients attending antiretroviral treatment clinic follow up at Mekelle hospital, Ethiopia: a cross sectional study. BMC Res Notes.

[9] Yonah G, Fredrick F, Leyna G (2014). HIV serostatus disclosure among people living with HIV/AIDS in Mwanza, Tanzania. AIDS Res Ther.

[10] Elizabeth S Kiula DJD, and Sia E Msuya. Predictors of HIV serostatus disclosure to partners among HIV-positive pregnant women in Morogoro, Tanzania. BioMed Central Public Health. 2013;23:433. 10.1186/1471-2458-13-433 PMC3668140 23641927

[11] Mlambo M, Peltzer K (2011). HIV sero-status disclosure and sexual behaviour among HIV positive patients who are on antiretroviral treatment (ART) in Mpumalanga, South Africa. J Human Ecol.

[12] Arrivé E, Dicko F, Amghar H, Aka AE, Dior H, Bouah B (2012). HIV status disclosure and retention in care in HIV-infected adolescents on antiretroviral therapy (ART) in West Africa. PLoS One.

[13] Seid M, Wasie B, Admassu M. Disclosure of HIV positive result to a sexual partner among adult clinical service users in Kemissie district, northeast Ethiopia. Afr J Reprod Health. 2012;16(1):97–104. 22783673

[14] Moher David, Liberati Alessandro, Tetzlaff Jennifer, Altman Douglas G. (2009). Preferred Reporting Items for Systematic Reviews and Meta-Analyses: The PRISMA Statement. PLoS Medicine.

[15] Stroup DF, Berlin JA, Morton SC, Olkin I, Williamson GD, Rennie D (2000). Meta-analysis of observational studies in epidemiology: a proposal for reporting. Jama..

[16] Moola S, Munn Z, Tufanaru C, Aromataris E, Sears K, Sfetcu R, Currie M, Qureshi R, Mattis P, Lisy K. Systematic reviews of etiology and risk. Joanna Briggs Institute Reviewers Manual. The Joanna Briggs Institute. 2017. 10.1097/XEB.0000000000000064 26262566

[17] Munn Z, Moola S, Lisy K, Riitano D, Tufanaru C (2015). Methodological guidance for systematic reviews of observational epidemiological studies reporting prevalence and cumulative incidence data. Int J Evidence Based Healthc.

[18] Borenstein M, Hedges LV, Higgins JP, Rothstein HR (2010). A basic introduction to fixed-effect and random-effects models for meta-analysis. Res Synth Methods.

[19] Higgins JP, Thompson SG, Deeks JJ, Altman DG (2003). Measuring inconsistency in meta-analyses. BMJ: Bri Med J.

[20] Gadisa T, Tymejczyk O, Kulkarni SG, Hoffman S, Lahuerta M, Remien RH (2017). Disclosure history among persons initiating antiretroviral treatment at six HIV clinics in Oromia, Ethiopia, 2012–2013. AIDS Behav.

[21] Kassahun G, Tenaw Z, Belachew T, Sinaga M. Determinants and Status of HIV Disclosure among Reproductive Age Women on Antiretroviral Therapy at Three Health Facilities in Jimma Town, Ethiopia, 2017. Health Sci J. 2018;12(2):258–263.

[22] Deribe K, Woldemichael K, Njau B, Yakob B. Gender difference in HIV status disclosure among HIV positive service users. East Afr J Public Health. 2009;6(3):251–256. 20803914

[23] Natae S, Negawo M (2016). Factors affecting HIV positive status disclosure among people living with HIV in west Showa zone, Oromia, Ethiopia; 2013. Abnorm Behav Psychol.

[24] Geremew TD, Nuri RA, Esmael JK (2018). Sero status disclosure to sexual partner and associated factors among adult HIV positive patients in bale zone hospitals, Oromia region, Ethiopia: institution based cross-sectional study. Open J Epidemiol.

[25] Firehiwot B Bewket T FA. Prevalence Of Sero Status Disclosure To Sexual Partner And Associated Factors Among Clients Attending Antiretroviral Treatment Clinic At University Of Gondar Referral Hospital,Northwest Ethiopia, Gondar, 2016. 2017. 2016.

[26] Alemayehu D, Tadess S, Adefris M, Birhanu Z (2014). HIV serostatus disclosure and associated factors among HIV positive pregnant women attending antenatal care services in northwest of Ethiopia. Int J Infect Control.

[27] Erku TA, Megabiaw B, Wubshet M. Predictors of HIV status disclosure to sexual partners among people living with HIV/AIDS in Ethiopia. Pan Afr Med J. 2012;13. PMC3567411 23396625

[28] Alemayehu M, Aregay A, Kalayu A, Yebyo H (2014). HIV disclosure to sexual partner and associated factors among women attending ART clinic at Mekelle hospital, northern Ethiopia. BMC Public Health.

[29] Deribe B, Ebrahim J, Bush L (2018). Outcomes and factors affecting HIV status disclosure to regular sexual partner among women attending antiretroviral treatment clinic. J AIDS Clin Res.

[30] Gari T, Habte D, Markos E. HIV positive status disclosure among women attending art clinic at Hawassa University Referral Hospital, South Ethiopia. East Afr J Public Health. 2010;7(1). 21413581

[31] Sendo EG, Cherie A, Erku TA. Disclosure experience to partner and its effect on intention to utilize prevention of mother to child transmission service among HIV positive pregnant women attending antenatal care in Addis Ababa, Ethiopia. BMC Public Health. 2013;13(1):765–771. 10.1186/1471-2458-13-765 PMC3844305 23957627

[32] Dessalegn NG, Hailemichael RG, Shewa-amare A, Sawleshwarkar S, Lodebo B, Amberbir A (2019). HIV disclosure: HIV-positive status disclosure to sexual partners among individuals receiving HIV care in Addis Ababa, Ethiopia. PloS One.

[33] Reda AA, Biadgilign S, Deribe K, Deribew A (2013). HIV-positive status disclosure among men and women receiving antiretroviral treatment in eastern Ethiopia. AIDS Care.

[34] Kebede Deribe Kassaye WL, Dejene Y (2005). Determinants and outcomes of disclosing HIV-sero positive status to sexual partners among women in Mettu and Gore towns, Illubabor zone Southwest Ethiopia. Ethiop J Health Dev.

[35] Kebede D, Wassie L, Yismaw D (2005). Determinants and outcomes of disclosing HIV-sero positive status to sexual partners among women in Mettu and Gore towns, Illubabor zone Southwest Ethiopia. Ethiop J Health Dev.

[36] Biadgilign S, Deribew A, Amberbir A, Deribe K (2008). Adherence to highly active antiretroviral therapy and its correlates among HIV infected pediatric patients in Ethiopia. BMC Pediatr.

[37] Vaz LM, Maman S, Eng E, Barbarin OA, Tshikandu T, Behets F (2011). Patterns of disclosure of HIV status to infected children in a sub-Saharan African setting. J Dev Behav Pediatr.

[38] Salami A, Fadeyi A, Ogunmodede J, Desalu O (2011). Status disclosure among people living with HIV/AIDS in Ilorin, Nigeria. West Afr J Med.

[39] Lugalla J, Yoder S, Sigalla H, Madihi C (2012). Social context of disclosing HIV test results in Tanzania. Cult Health Sex.

[40] Albert A, Salako OOSaOEA. Disclosure of HIV Status among Clients Accessing Care at A Tertiary Health Facility in Sagamu, Southwestern Nigeria. Curr Res J Biol Sci. 2016;8(2):18–23.

[41] Li Li, Luo Sitong, Rogers Benjamin, Lee Sung-Jae, Tuan Nguyen Anh (2016). HIV Disclosure and Unprotected Sex Among Vietnamese Men with a History of Drug Use. AIDS and Behavior.

[42] Gadisa T, Tymejczyk O, Kulkarni SG, Hoffman S, Lahuerta M, Remien RH (2016). Disclosure history among persons initiating antiretroviral treatment at six HIV clinics in Oromia, Ethiopia, 2012–2013. AIDS Behav.

[43] Pamela Bachanas P, MA, Amy Medley, PhD, MPH, Sherri Pals, PhD, MS, Daniel Kidder, PhD, MS, Gretchen Antelman, PhD, Irene Benech, MD, Nicolas Deluca, PhD, MA, Harriet Nuwagaba-Biribonwoha, PhD, MBChB, Odylia Muhenje, MA, Peter Cherutich, MD, MPH, Pauline Kariuki, BA, RN, Frieda Katuta, BSc, RN, and May Bukuku, MD, MPH,. Disclosure, Knowledge of Partner Status, and Condom Use Among HIV-Positive Patients Attending Clinical Care in Tanzania, Kenya, and Namibia. AIDS Patient Care STDS. 2007;27(7):425–435. 10.1089/apc.2012.0388 PMC3704112 23829332

[44] Kidane A. Sarkoa B, Meridith Blevinsc, Aimalohi A, Ahonkhaia E, Carolyn M, Audeta B, Troy D, Moona D, Usman I, Gebib F, Ahmed M, Ganag C, William Westera E, Sten H, Vermund H and Muktar H. Aliyu. HIV status disclosure, facility-based delivery and postpartum retention of mothers in a prevention clinical trial in rural Nigeria. R Soc Trop Med Hyg. 2017;9:243–251. 10.1093/inthealth/ihx023 PMC5881261 28810669

[45] Conserve Donaldson F., King Gary, Dévieux Jessy G., Jean-Gilles Michèle, Malow Robert (2014). Determinants of HIV Serostatus Disclosure to Sexual Partner Among HIV-Positive Alcohol Users in Haiti. AIDS and Behavior.

[46] LMSd S. The family’s role as a support network for people living with HIV/AIDS: a review of Brazilian research into the theme. Ciência Saúde Coletiva. 2014;20(4):1109-18. 10.1590/1413-81232015204.17932013 25923622

